# Airborne transmission of SARS-CoV-2 disease (COVID-19)

**DOI:** 10.2217/fvl-2021-0324

**Published:** 2022-03-01

**Authors:** Mohammad K Parvez, Shama Parveen

**Affiliations:** ^1^Department of Pharmacognosy, King Saud University College of Pharmacy, Riyadh, 11451, Saudi Arabia; ^2^Centre for Interdisciplinary Research in Basic Sciences, Jamia Millia Islamia Central University, New Delhi, 110025, India

**Keywords:** aerosol, airborne transmission, SARS-coronavirus-2, COVID-19, droplets, SARS-CoV-2

Since its first appearance in 2019, SARS-CoV-2 disease (COVID-19) has continued to cause massive devastation, worldwide [1,2]. Emergence of its aggressive mutant strains or genetic variants in Brazil, the USA, the UK, South Africa, India or elsewhere and their subsequent spread in other countries has further worsened the crisis [3]. SARS-CoV-2 generally manifests into fever, cough, nausea, headache, hypoxia or diarrhea and may progress to mild-to-severe pneumonia and death [1]. Fortunately, we have acquired an in-depth knowledge on the epidemiology, manifestations, pathobiology, prevention and treatments modalities of SARS-CoV-2 as well as its ‘variants of concern’. In the absence of specific therapeutics, several repurposed drugs are currently under advanced phases of clinical trials or approved for emergency use [4]. Notably, within one and half years of this pandemic, some of the leading vaccines have been granted approval for mass vaccination, worldwide.

Viral transmission rates are often higher in dense than in sparse populations, where social contacts greatly enhance their spread. The ‘human-to-human’ direct transmission of SARS-CoV-2 through multiple modes, such as naso/oropharyngeal droplets, aerosols and fomites have been confirmed [1]. Therefore, as advised by the international health regulatory authorities, wearing masks, maintaining physical distancing and hand or surface sanitization would limit the risk of infection. In addition, recent data on detection of SARS-CoV-2 in a COVID-19 patient’s stool and several water sources, suggest its plausible waterborne spread through fecal–oral route [5,6].

## Contagious droplets & aerosols

Mucous droplets exhaled due to normal breathing, sneezing or coughing form multiphase turbulent gas clouds or puffs which can travel long distances and eventually evaporate, leaving aerosol particles (<5 μm) suspended in the air for several hours [7–9]. Micro droplets and aerosols are differentially defined using a size threshold of 100 μm [10], which differentiates their aerodynamics, inhalable ability and the efficacy of intervention strategies. Generally, viruses in expelled droplets (>100 μm) fall to the ground in seconds and can be sprayed onto nearby individuals or surfaces within 6 feet of the source. In contrast, viruses in comparatively smaller aerosols (<100 μm) can remain suspended in the air as particulate matter (PM) for several hours, which can be inhaled efficiently. While a single cough can release hundreds of such droplets, each containing millions of virus particles, a single sneeze may generate up to 40,000 micro droplets at speeds of 50–200 miles per hour [11]. Aerosolized micro droplets, therefore, can travel 6 feet or further before settling on the ground or surfaces, making them infectious for hours or days. However, in experimental conditions, the contagious droplets and aerosols inside a turbulent puff cloud have been shown to travel up to 26 feet and keep infectious viruses up to 3 h [12].

## Airborne or atmospheric transmission of SARS-CoV-2

Studies of influenza virus, rhinovirus, respiratory syncytial virus, varicella-zoster virus and measles virus-infected breath- and cough-generated aerosols (<4.7 mM) have shown presence of viral RNA in hospital-room air samples [13,14]. Very little data are available on SARS-CoV-1 air sampling during the 2002–2003 pandemic [15,16]. For COVID-19, SARS-CoV-2-loaded respiratory droplets (<5 μm) have been demonstrated to travel up to a 6 feet distance in air [17,18]. Further studies of hospital air samples in China and North America have detected SARS-CoV-2 RNA in aerosols [17,19,20].

There is growing evidence that inhalation of SARS-CoV-2 represents a major transmission route for COVID-19 [21,22]. Recent studies have shown that SARS-CoV-2 can be transmitted by infected persons over long distances or periods of time in enclosed or indoor environments such as homes, apartments, offices, schools, hostels, workplaces, washrooms, hospitals, restaurants, airports, train stations and public transport systems [23–26]. According to the CDC guidelines, circumstances under which airborne transmission of SARS-CoV-2 appears to have occurred include: enclosed spaces where an infectious person either exposes the susceptible population at the same time or shortly after the source leaves the space; prolonged exposure to respiratory particles, often generated through speaking, shouting, singing, coughing, sneezing, yawning etc., which increase the concentration of suspended droplets, and inadequate ventilation or air handling that allows a build-up of suspended small respiratory droplets and particles in the air space [27]. Nonetheless, the overall evidence regarding the airborne transmission of SARS-CoV-2 hitherto remains inconclusive [9,28,29]. Though airborne spread of COVID-19 within closed environments is plausible, there has been no evidence for detection of SARS-CoV-2 in indoor air. While a recent investigation of the dry air of COVID-19 patient rooms has resulted in a negative test [30], the presence of SARS‐CoV‐2 in the indoor air at 3 m from the patients through contaminated droplets has been reported [31].

Furthermore, an Italian study has revealed the accelerated transmission of SARS-CoV-2 because of ‘air pollution’ measured with days exceeding the set limits (PM_10_) [32]. Therein, as compared with the high-wind coastal cities, low-wind hinterland cities with average set limits had significantly high transmission rates. This study suggested the accelerated ‘polluted air-to-human’ transmission dynamics of SARS-CoV-2 [33]. Moreover, the community spread of such viral diseases has been also linked to the inhalation of aerosolized or splattered infectious particles. Previously, several clusters of airborne transmission of SARS-CoV-1 have been reported, including transmission of the virus to passengers from an infected person located seven rows apart in an aircraft [34], transmission among guests sharing the same floor of a hotel [35] and spread among hundreds of residents of a housing society due to a faulty drainage system [36]. Taken together, these data suggest a risk of environmental or atmospheric contamination of SARS-CoV-2 and its potential airborne transmission.

Very recently, an indoor safety guideline has been proposed based on mathematical models of the airborne transmission, applying the product of the number of occupants and their exposure time in a closed space [37]. Therein, by synthesizing available data from the best-characterized indoor spreads with droplet-size distributions, an infectious dose on the order of ten aerosol-borne SARS-CoV-2 has been estimated. This has included case studies for classrooms and nursing homes, and a provision of a spreadsheet to facilitate use of this guideline.

## Conclusion & future perspective

In the early stages the COVID-19 pandemic, ‘dirty surfaces’ were greatly warned as the potential sources of its spread. Later, as the pandemic progressed, there emerged multiple reports of its outbreak in ‘crowded spaces’, especially in closed or poorly-ventilated places. Nonetheless, while the epidemiological data has indicated that most of COVID-19 spread occurs through close contacts, its atmospheric or airborne transmission can occur under special circumstances. The existing interventions and preventive measures to control its potential airborne spread include physical distancing, use of good quality masks, environmental hygiene, avoidance of crowded outdoor spaces and improving adequate ventilation of indoor spaces. A crucial factor is use of high quality masks with a good fit, which could effectively filter the infectious aerosols as well as restrict their way around any gaps between mask and nose. While risk-assessment tools have been developed for SARS-CoV-2 transmission and the airborne spread of its aggressive variants is globally recognized, no specific safety guideline has been issued by the world health authorities. Following the genetic evolutionary rule, SARS-CoV-2 will continue to mutate into more or less aggressive strains. Such new variants would establish their circulation in the general population despite the acquired ‘herd immunity’ or mass vaccination. At the present time, though there seems to be no need to devise special engineering controls to protect the general community, it would be important to estimate the probability of infectious virus-loaded aerosols, toward preventing further transmission of COVID-19.
